# Diseases of the Negro Population

**Published:** 1845-02

**Authors:** Daniel Drake


*Diseases of the Negro Population.* By Daniel Drake, M.D., in a letter to Rev. Mr. Pinney:

MEDICAL INSTITUTE OF LOUISVILLE, Nov. 15, 1844.

DEAR SIR: Since our interview in Cincinnati, I have been so much engaged in entering on my duties for the winter, as to be unable till now, to comply with your request, for some notice of the diseases of the colored population of the South and West. As I told you then, my inquiries were chiefly made in Alabama, Mississippi, and Louisiana, in the spring and summer of 1843 and '44. Of the diseases of which I am about to mention, I witnessed most of the varieties, but the greater and better part of my information was derived from conversations with physicians, planters, and overseers, carefully noted down at the time. By referring to these, I give you the following statement:

1. Many infants die of trismus, or lock-jaw, when they are but a few days old; after that early age, convulsions, and summer sickness, (cholera infantum,) and worms, carry off quite a number.

2. They are liable to measles and scarlet fever, both of which were prevailing (but especially the former) on many plantations which I visited; which diseases seem to be as fatal to them as to the whites.

3. Scrofula or king's-evil is of frequent occurrence; and consumption, or cachexia Africana, as it has been called, is prevalent and always fatal.

4. On many plantations the strange habit prevails of eating dirt or clay, the common soil of the fields, particularly that of the Mississippi bottoms, producing serious and fatal diseases. I was told of one estate in South Alabama, on which fourteen slaves had died from this cause, and visited another in Louisiana, on which I saw nearly half that number unable to work from the same practice.

5. A disease of the heart, conjectured to arise from dirt-eating, destroys quite a number. I met with several cases, and heard of a plantation on Red River, where more than thirty died from this malady.

6. Tetanus or lock-jaw from wounds, is extremely common and almost uniformly fatal. Some cases occur without previous wounds. A physician in Alabama told me he had, in fifteen years, met with at least fifty cases, nearly all colored people, and all but one mortal. I met with several young physicians in the smaller towns, who had, respectively, met with more cases than have occurred in Cincinnati from its first settlement.

7. Diarrhœa and dysentery, of frequent occurrence, are often fatal.

8. Where the cholera was epidemic, in 1832, '33, and '34, it swept off great numbers; was more destructive, in fact, to the colored than the white people of the Southwest.

9. Epidemic erysipelas, or black tongue, has prevailed on many plantations within the past year. I was told of one, in Mississippi, on which seven had died of it.

10. The colored people are not proof against the cause of yellow fever, but as they are not numerous in the cities and towns, where only it prevails, the mortality from this disease is not great.

11. Acute inflammations of the lungs are among the most destructive diseases of the colored population. These are catarrh, croup, bronchitis, pleurisy, and pneumonia, or inflammation of the substance of the lungs, which is the most frequent and fatal of the whole. These maladies often destroy life in a few days; but sometimes the patient recovers with his lungs rendered permanently unsound. I saw many cases of this kind. This group of diseases, produced by changes of weather in winter and early spring, occasions more deaths than any other, except the next.

12. Intermittent, and remittent fevers; simple, and malignant or congestive, are the greatest outlets of human life among the people of whom I am speaking. They return every year in the latter part of summer and in autumn, and one attack is no security against another. When they do not prove fatal, they leave behind them diseases of the spleen and dropsy. In the following winter those who were down in the autumn, are tender and often die of inflammation of the lungs.

In addition to the diseases I have named, others occur now and then, with considerable frequency, of which I may mention rheumatism, epilepsy, colic, hysteria, and several infirmities peculiar to women.

From this catalogue you will perceive that the colored population of the Southwest are by no means exempt from a variety of formidable diseases. As we come further north tetanus and autumnal fever get less, but consumption and inflammations of the lungs increase. All over the region of which I have spoken, the greatest part of the practice of every country physician is among the colored people. A gentleman in Louisiana told me that he received a salary of $1,200 a year for attending on a single plantation. From all I have read and heard on the diseases of Liberia, my impression is, that if half the colored population of a Southwestern plantation were sent to the colony, they and their descendants, in ten years, would number more than those left behind.

With great respect, I remain, dear sir, your obedient servant,

DANIEL DRAKE.

Mr. Pinney.

*Col. Herald.*

